# Multitech-Based Study on Medicinal Material Basis and Action Mechanism of Herbal Formula Xian-Ling-Gu-Bao Capsule in Treatment of Osteoarthritis

**DOI:** 10.1155/2022/6986372

**Published:** 2022-09-06

**Authors:** Xiaowen Wu, Shuai Sun, Xiaoyi Wu, Zengxian Sun

**Affiliations:** Department of Pharmacy, The Affiliated Lianyungang Hospital of Xuzhou Medical University (The First People's Hospital of Lianyungang), Lianyungang 222000, China

## Abstract

Currently, osteoarthritis (OA) is thought to be the most prevalent chronic joint disease worldwide. The epidemiology of this disorder is complex, and the treatment is challenging. Xian-Ling-Gu-Bao (XLGB) capsule, a herbal compound preparation, is widely used for the treatment of bone disorders, including OA. Although its efficacy and safety have been demonstrated in clinical trials and practice, the underlying medicinal constituents and mechanism have not been clearly elucidated. Therefore, this study aimed to explore the medicinal constituents and mechanism of XLGB for OA treatment. The phytochemical constituents in XLGB capsule were detected by liquid chromatography-mass spectrometry (LC-MS), the medicinal constituents and therapeutic mechanism for OA treatment were deduced by network analysis, and the deduced mechanism was validated by *in vitro* experiment. As a result, a total of 55 constituents were detected in XLGB extract, in which 16 constituents were screened out for target collection. Based on the analysis of target profile, XLGB targets showed a high degree of similarity with OA targets. Network analysis revealed that XLGB had a holistic effect of multiple active constituents on multiple targets and pathways. The core targets of XLGB were presumed to be MAPKs, PI3K, AKT, BCL2, RELA, TNF, NOS2, and so on, and the mechanism was speculated to mainly inhibit chondrocyte apoptosis and inflammatory response through JNK and PI3K/AKT/NF-*κ*B signaling cascades. Finally, *in vitro* study confirmed that XLGB extract protected ATDC5 cells against lipopolysaccharide- (LPS-) induced apoptosis and inflammatory response, and these effects were supposed to be involved in the inhibition of JNK and PI3K/AKT/NF-*κ*B pathways. Our study could provide a scientific basis for further research and clinical use of XLGB capsule.

## 1. Introduction

Osteoarthritis (OA) is characterized by joint structural change, including articular cartilage degradation, subchondral bone remodeling, and periarticular muscle weakness [1]. The cardinal signs include pain, stiffness, and loss of function, thus leading to considerable impairment of quality of life [2]. It has become the most prevalent degenerative arthritis, estimated to affect 10% of men and 18% of women over 60 years of age over the world [3]. The total incidence of knee arthritis in the population over 65 years old is up to 50% in China [4]. With the acceleration of population aging, the incidence of OA shows a trend of steady growing. Therefore, this syndrome has been widely regarded as an increasing burden, leading to a substantial strain on the healthcare system.

The pathophysiology of OA is complex and involves multiple joint tissues, including cartilage, subchondral bone, and synovium. Thus, OA is now considered a whole joint disease [5]. This disease represents a dynamic imbalance between the repair and destruction of joint tissues. Many factors, such as mechanical, metabolic, and inflammatory lesions, play key roles in disease pathogenesis [5–7]. Recommendations for OA treatment are often separated into nonpharmacological, pharmacological, and surgical interventions [8]. Nonpharmacological interventions often include patient education, exercise, and weight loss, which represent a core component of OA management [6]. Joint surgery should be considered for patients with severe symptoms and failed conservative therapy [7]. Existing pharmaceutical therapies are offered to reduce the symptoms, primarily joint pain, whereas these approaches are hardly capable of acting on the pathophysiologic mechanisms underlying the disease. First-line therapies include topical nonsteroidal anti-inflammatory drugs (NSAIDs) and oral paracetamol. Topical NSAIDs provide almost equivalent efficacy but better safety profile to oral NSAIDs; however, these drugs still provide only minimal improvement in joint function [9–12]. Injection of intra-articular corticosteroids is recommended by the American College of Rheumatology and the Osteoarthritis Research Society International when a feeble outcome is achieved from analgesics, but the evidence for its efficacy remains unclear [13]. In a word, current pharmaceutical interventions are merely symptomatic and are also characterized by relatively small effect sizes or/and uncertainty of long-term safety [14].

Herbal remedy, often manifesting the combination of different herbs, could theoretically target multiple targets/pathways due to the abundant bioactive phytochemicals in herbs. Therefore, herbal remedy secures a place in the treatment of complex and chronic diseases since ancient times and accordingly has a long tradition in the treatment of OA. Xian-Ling-Gu-Bao (XLGB) is a well-known Chinese herbal recipe, and its capsule dosage form has been clinically used for the treatment of OA, osteoporosis, aseptic osteonecrosis, and bone fracture since official approval by the Chinese State Food and Drug Administration (SFDA) [15, 16]. It consists of 6 species of medicinal herbs: *Epimedium brevicornum* Maxim (EbM, 70%), *Dipsacus asper* Wall. ex DC (DaW, 10%), *Cullen corylifolium* (L.). Medik. (CcM, 5%), *Salvia miltiorrhiza* Bunge (SmB, 5%), *Anemarrhena asphodeloides* Bunge (AaB, 5%), and *Rehmannia glutinosa* (Gaertn.) DC. (RgG, 5%). According to the Chinese medicine theory, the herbs in XLGB have the effects of nourishing liver and kidney, strengthening bone and muscle, dispelling wind and eliminating dampness, eliminating stagnation, and removing blood stasis; thus, this prescription is very suitable for the treatment of bone diseases. A latest randomized controlled trial demonstrated that XLGB significantly reduced pain and improved knee and hand function in OA patients over a 6-month period [16]. Besides, several studies have also investigated the mechanism of XLGB against osteoporosis [15, 17, 18], whereas the research involved in OA remains limited. Moreover, although effort has also been made to explore the chemical constituents, quality control, and pharmacokinetics of XLGB [19–21], the pharmacological mechanism of XLGB against OA has not been clearly elucidated due to the complex nature of herbal medicine. Interestingly, network pharmacology is an emerging technique widely used for investigating the mechanism of traditional herbal drugs in the past decade [22–24]. Therefore, herein we integrated different approaches, including phytochemical analysis, network pharmacology, and *in vitro* experiment, to cope with this challenge. Firstly, the phytochemical constituents were detected in the extract of XLGB capsules, then network analysis was performed to deduce the active constituents and action mechanism, and finally *in vitro* study on murine ATDC5 cells was conducted to validate the deduction.

## 2. Material and Methods

### 2.1. Phytochemical Analysis of XLGB Samples

XLGB capsules were purchased from Sinopharm Tongjitang (Guizhou) Pharmaceutical Co., Ltd. (Guiyang, China). The content of XLGB capsules was weighed and then dispersed in 60% (v/v) aqueous ethanol (1 : 10, w/v). The mixture was subjected to ultrasonication extraction for 30 min at room temperature. After centrifugation, the supernatant was collected and then filtered through a 0.22 *μ*m nylon membrane filter. A total of 16 reference standards purchased from Chengdu MUST Bio-Technology Co., Ltd. (Chengdu, China), including caffeic acid, ferulic acid, loganic acid, magnoflorine, sweroside, timosaponin BII, salvianolic acid B, icariin, epimedin C, chlorogenic acid, psoralen, neochlorogenic acid, icaritin, bavachin, psoralidin, and tanshinone IIA, were dissolved in methanol for LC-MS analysis for qualitative identification.

Sample analysis was carried out on an Agilent 6550 liquid chromatography coupled with a quadrupole time-of-flight mass spectrometry (LC-Q/TOF-MS) system. Chromatographic separation was performed on a Sepax GP-C18 column at 35°C, and the mobile phase consisted of 0.1% formic acid (A) and acetonitrile (B) with a flow rate of 200 *μ*L/min. The gradient elution was set as follows: 0–5 min, 15%–25% B; 5–10 min, 25%–40% B; 10–15 min, 40%–50% B; 15–20 min, 50%–60% B; 20–25 min, 60%–75% B; 25–30 min, 75%–85% B; 30–35 min, 85%–90% B. Both positive and negative modes of electrospray ionization (ESI) were performed under optimal parameters: ion spray voltage 3500 V for positive mode and 3000 V for negative mode; vaporizer temperature 280°C; sheath gas pressure 50 psi; capillary temperature 320°C; and auxiliary gas pressure 15 psi. The full scan covered the mass range from 100 to 1000 Da. Fragment ions of analytes were obtained from MS/MS fragmentation at different collision energy. Phytochemical constituents were identified by matching their retention times, molecular ions, and product ions with reference standards and literature data, as well as predicting according to their fragmentation patterns with an accuracy error threshold fixed at 10 ppm.

### 2.2. Constituent Screening and Target Collection

Oral bioavailability (OB) is one of the most commonly used parameters for screening candidate compounds with the potential to be further developed into drugs [25], while drug-likeness (DL) represents how the pharmaceutical properties of a compound correspond in the majority of available drugs [26]. In this work, OB ≥ 30% and DL ≥ 0.18 were set as a threshold for screening the candidate constituents in XLGB capsule. The abundant phytochemicals in XLGB have an implication that its efficacy could be derived from acting on multiple targets. Herein, specialized databases, including TCMSP (Traditional Chinese Medicine Systems Pharmacology Database and Analysis Platform, https://tcmsp-e.com/tcmsp.php) and STITCH (https://stitch.embl.de/), were used to search the putative targets of constituents in XLGB. Furthermore, as a supplement, a large-scale text mining of PubMed was performed to manually extract the relevant targets from literature. The collected targets were transformed into their official symbols based on the UniProtKB database (https://www.uniprot.org/) with the species limited to “Homo sapiens.” Meanwhile, the target information of OA, including “Symbol” and “Score” items, was exported from the human gene database GeneCards (https://www.genecards.org/).

### 2.3. Analysis of Target Profile and Herbal Contribution

Different compounds could have the same potential targets; thus, the repetitive targets were merged. Meanwhile, the repetition number of each target was counted and then normalized. Consequently, the target quantitative profile of XLGB was established. Likewise, the “Score” values of OA targets were normalized through being divided by the maximum value. After normalization, the target quantitative profile of OA was also established. The target intersection between XLGB and OA was identified and then depicted by Venn diagram. The target coverage and contribution of each herb were analyzed. The relevance of target profile between XLGB and OA was evaluated by hierarchical cluster analysis (HCA) performed in Heml (Ver. 1.0).

### 2.4. Analysis of Network Interaction and Pathway Enrichment

To explore the interaction between XLGB constituents and their potential targets, an integrated network consisting of herb-constituent interaction, constituent-target interaction, and target-disease interaction was constructed in Cytoscape software (Ver. 3.7). In the network, the degree value of each node was calculated using the NetworkAnalyzer, and then visually characterized with its size. Based on the degree values, top targets and constituents were extracted and analyzed. Subsequently, the network of protein-protein interaction (PPI) was constructed by importing XLGB-OA intersection targets into the STRING database (https://www.string-db.org/). In the PPI network, the degree values of targets were visually characterized by node sizes, and the top targets were extracted to generate a new subnetwork. Along with PPI analysis, gene ontology (GO) enrichment was also performed, and then the top functional annotations regarding biological processes, molecular functions, and cellular components were analyzed.

Enrichment analysis of Kyoto Encyclopedia of Genes and Genomes (KEGG) pathways was performed to explore the relevant pathways and embedded targets. *p* value was given for each KEGG pathway, and smaller *p* value suggested greater enrichment. Based on the given parameters, the top pathways were extracted and analyzed for deciphering the action mechanism of XLGB against OA.

### 2.5. Experimental Validation

#### 2.5.1. Cell Culture and Treatment

Murine chondrogenic cell line ATDC5 cells (ATCC; Manassas, VA, USA) were cultured in a complete RPMI-1640 medium (Gibco, Grand Island, NY, USA) containing 10% fetal bovine serum (HyClone; Logan, UT, USA), 100 U/mL penicillin, and 100 *μ*g/mL streptomycin (Gibco; Grand Island, NY, USA) in a humidified incubator containing 95% air and 5% CO_2_ at 37°C. When cell confluence achieved 80%, cells were induced by 5 *μ*g/mL lipopolysaccharide (LPS) (Sigma-Aldrich; St. Louis, MO, USA) for 12 h. The content of XLGB capsules was weighed and then dispersed in 60% (v/v) aqueous ethanol (1 : 10, w/v). The mixture was subjected to ultrasonication extraction for 30 min at room temperature. After centrifugation, the supernatant was evaporated at 45°C under reduced pressure. Subsequently, the solution was made up to the final concentration of 0.8 g/mL (equivalent to the weight of the content of XLGB capsule). For drug administration, the extracts of XLGB capsules were dissolved in dimethyl sulfoxide (DMSO) to a concentration of 100 mg/mL. The cells were treated with different concentrations of XLGB extracts for 12 h before LPS stimulation. The final concentrations of DMSO in the cell cultures were less than 0.1%, and thus the effect of DMSO is negligible. Therefore, no DMSO was included in the control group.

#### 2.5.2. Cell Viability Assay

Cell viability of ATDC5 cells was evaluated by using Cell Counting Kit-8 (CCK-8) assay. Cells were seeded into 96-well plates at a density of 5 × 10^3^ cells/well. After treatments with XLGB extracts and/or LPS, CCK-8 solution (Sigma-Aldrich; St. Louis, MO, USA) was added, followed by additional incubation at 37°C for 1 h. The absorbance at 450 nm was read using a Microplate Reader (Bio-Rad; Hercules, CA, USA).

#### 2.5.3. Cell Apoptosis

Cell apoptosis was evaluated by using Annexin V-FITC/PI apoptosis detection kit (Invitrogen; Carlsbad, CA, USA). Briefly, after treatment, cells were collected and washed with phosphate-buffered saline (PBS). Then, cells were stained using a kit solution in the dark and then analyzed by a flow cytometer (Beckman Coulter, Miami, FL, USA). The obtained data were resolved by FlowJo software (Tree Star; San Carlos, California, USA).

#### 2.5.4. Enzyme-Linked Immunosorbent Assay (ELISA)

After the indicated treatment, culture supernatant of ATDC5 cells was collected from 6-well plates, and the concentrations of TNF-*α*, IL-1*β*, IL-6, and iNOS were, respectively, measured by using a mouse ELISA Kit (Solarbio, Beijing, China) according to the manufacturer's instructions.

#### 2.5.5. Western Blot

Total proteins of ATDC5 cells were isolated using RIPA lysis buffer (Beyotime Biotechnology; Shanghai, China) supplemented with protease inhibitors (Roche, Basel, Switzerland). Equal amount of protein samples (40 *μ*g) was subjected to SDS-PAGE and then transferred onto polyvinylidene fluoride (PDVF) membranes (Millipore; Bedford, MA, USA). The membranes were incubated with primary antibodies against Bcl-2, Bax, caspase-3, cleaved caspase-3, JNK, p-JNK, PI3K, phospho (p)-PI3K, AKT, p-AKT, p65, p-p65, and *β*-actin (Abcam; Cambridge, UK) at 4°C overnight. After rinsing with Tris-buffered saline and tween (TBST), the membranes were incubated with a secondary antibody (HRP-conjugated Anti-Rabbit IgG). After rinsing again using TBST, protein signals were visualized using ultra signal chemiluminescence reagents (4A Biotech; Beijing, China) and then captured by Bio-Rad ChemiDoc™ XRS system (Bio-Rad; Hercules, CA, USA). The intensities of protein bands were analyzed using ImageJ software (NIH, Bethesda, MA, USA).

#### 2.5.6. Reverse Transcription-Quantitative PCR (RT-qPCR)

Total RNAs were isolated from ATDC5 cells using TRIzol reagent (Invitrogen, Carlsbad, CA, USA). cDNA was transcribed using SuperScript™ IV First-Strand Synthesis System (Invitrogen, Carlsbad, CA, USA). Thermal cycling and fluorescence detection were performed with SYBR™ Green PCR Master Mix (Applied Biosystems; Foster, CA, USA). Reaction cycle conditions were set as follows: 95°C for 10 min for initial denaturation, 40 cycles at 95°C for 15 s, 60°C for 20 s, and 72°C for 30 s. The relative mRNA expression of IL-1*β*, IL-6, TNF-*α*, and iNOS was quantified by the 2^−ΔΔCt^ method with normalization against *β*-actin. The primer sequences used in this study were listed in Table 1.

### 2.6. Statistical Analysis

Data were presented as mean ± standard deviation (SD) of three independent experiments. Statistical analysis was performed using GraphPad Prism 7 software (GraphPad; San Diego, CA, USA). *p*-values were calculated using one-way analysis of variance (ANOVA), and *p* < 0.05 was considered statistically significant.

## 3. Results

### 3.1. Phytochemical Constituent Analysis and Screening

**Figure 1:**
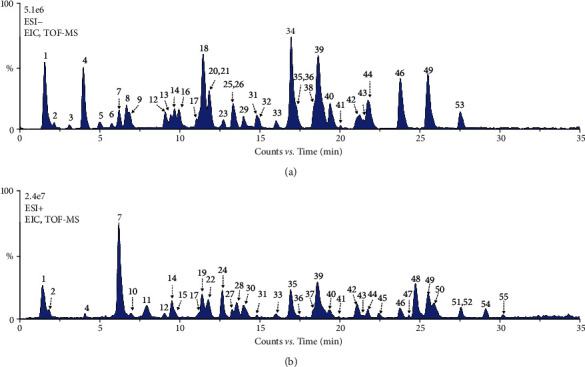
The representative EIC of XLGB samples obtained from LC-MS analysis with negative (a) and positive (b) ion modes (EIC, extracted ion chromatogram; XLGB, Xian-Ling-Gu-Bao; LC-MS, liquid chromatography coupled with mass spectrometry).

**Figure 2:**
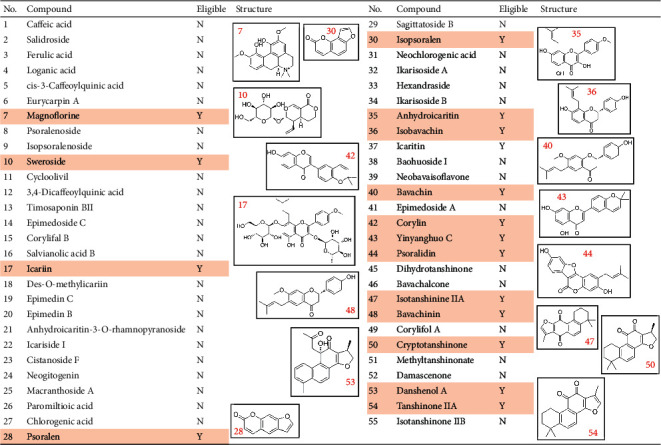
Phytochemical constituents identified in XLGB capsules and the structures of candidate compounds screened by OB and DL parameters. OB ≥ 30% and DL ≥ 0.18 were set as a threshold for screening the candidate constituents in XLGB capsule (XLGB, Xian-Ling-Gu-Bao; OB, oral bioavailability; DL, drug-likeness).

XLGB extracted samples were analyzed using LC-Q-TOF/MS with positive and negative ion modes. The generated extracted ion chromatograms (EIC) of XLGB samples are presented in Figure 1. Based on the reference standards, a total of 16 phytochemical constituents were identified by matching the retention time, high-resolution mass, and MS/MS fragment ions. The identities of another 39 phytochemical constituents were confirmed by comparing the information of retention time, high-resolution mass, and MS/MS fragments with relevant literature reports [19, 27]. In total, 55 main phytochemical constituents were identified in XLGB capsule samples, and detailed information is provided in Table S1. These constituents were mainly categorized as phenolic acids, flavonoids, coumarins, terpenoids, and tanshinones. After screening based on OB and DL, 16 of 55 phytochemicals were reserved and considered as potential active compounds, and their structures are provided in Figure 2. Among these compounds, icariin, magnoflorine, and Yinyanghuo C were the constituents of EbM, sweroside was a constituent of DaW, isotanshinone IIA, cryptotanshinone, danshenol A, and tanshinone IIA were the constituents of SmB, and anhydroicaritin was a constituent of AaB, while psoralen, isopsoralen, isobavachin, bavachin, corylin, psoralidin, and bavachinin were the constituents of CcM.

### 3.2. Target Collection and Analysis

**Figure 3:**
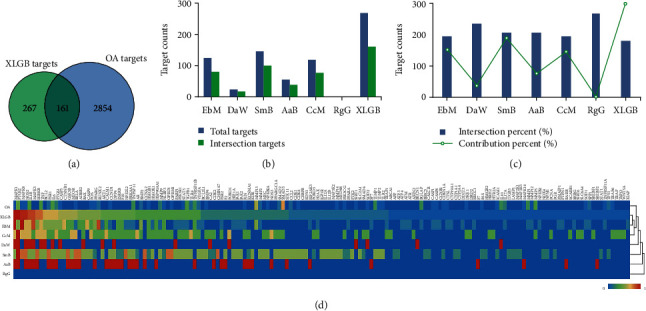
Target analysis between XLGB and OA. (a) Target intersection of XLGB and OA. (b) Target counts of individual herb in XLGB. (c) Target contribution of individual herb in XLGB. (d) Heatmap combined with HCA using the target profile of XLGB and OA (XLGB, Xian-Ling-Gu-Bao; OA, osteoarthritis; HCA, hierarchical cluster analysis).

Subsequently, these screened compounds were used for target collection. As a result, 16 candidate compounds yielded 267 targets after deleting duplicates. Meanwhile, a total of 2854 targets were collected from the GeneCards database for OA. As described in Material and Methods, the target profile was established for XLGB and OA, respectively. XLGB targets were mapped with OA targets, and then 161 interaction targets were generated (Figure 3(a)). As focusing on the individual herb in XLGB, the total targets and XLGB-OA intersection targets that each herb possessed were different. Among them, SmB possessed most of the total targets, followed by EbM, CcM, AaB, DaW, and RgG (Figure 3(b)). The condition of different herbs regarding intersection targets was similar to the above (Figure 3(b)). Yet it is worth noting that RgG yielded no target due to the fact that none of the detected phytochemicals belonged to it. After calculation, the target intersection rate between each herb and OA was all higher than 60%, except for RgG (Figure 3(c)). One herb possessing high intersection rate meant that it was more specific for the paired disease. Noticeably, DaW showed the highest target intersection rate with OA, although it yielded a small target count. In addition, the contribution percent of each herb varied distinctly. Among them, SmB contributed the most targets, followed by EbM, CcM, AaB, DaW, and RgG. HCA analysis was used to investigate the relevance of target profile between XLGB and OA. As shown in Figure 3(d), the horizontal axis represented targets, while the vertical axis represented OA, XLGB, and individual herb. By HCA analysis, the XLGB group was firstly merged into the same cluster with EbM, followed by CcM, and then into the same cluster with OA. These results suggested that EbM had a very similar target profile to XLGB. Besides, XLGB, EbM, and CcM presented a more similar target profile to OA compared with other herbs. To sum up, EbM and CcM contributed plenty of targets to XLGB, which were only next to SmB, and they also showed the most similar target profile to XLGB and OA. Although SmB possessed the most targets among the total of 6 herbs, it presented a relatively low similarity of target profile with OA.

### 3.3. Analysis of Network Interaction and Pathway Enrichment

**Figure 4:**
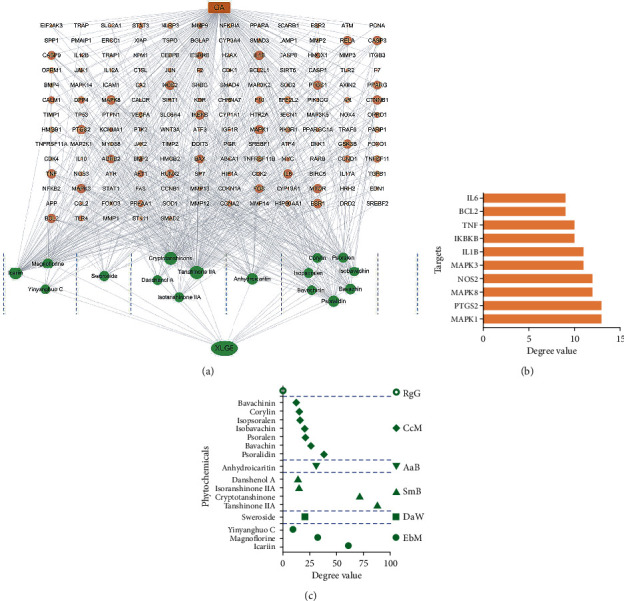
Interaction network and topological analysis. (a) XLGB-constituent-target-OA interaction network. (b) Top 10 targets screened out based on degree value. (c) Constituent sort based on herbal affiliation and degree value (XLGB, Xian-Ling-Gu-Bao; OA, osteoarthritis).

The interaction between 16 constituents and 161 targets was visualized by a network, in which the constituents with the same affiliation were clustered (Figure 4(a)). This diagram showed an interactive relationship between one constituent linking with multiple targets and one target pairing with multiple constituents. Based on the degree values, the top 10 targets were extracted as follows: MAPK1, PTGS2, MAPK8, NOS2, MAPK3, IL1B, IKBKB, TNF, BCL2, and IL6 (Figure 4(b)). Likewise, the constituents were also sorted based on their herbal affiliation and degree values (Figure 4(c)). Among them, tanshinone IIA and cryptotanshinone from SmB, icariin and magnoflorine from EbM, and psoralidin from CcM were the top 5 constituents at degree values.

**Figure 5:**
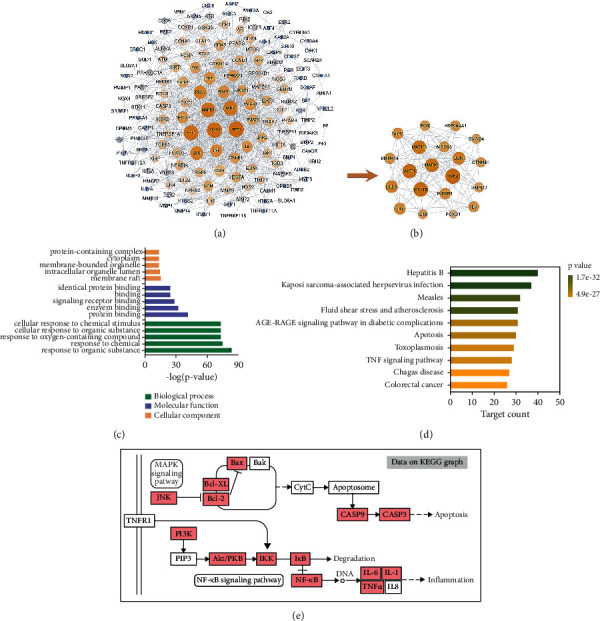
PPI network and enrichment analysis. (a) PPI network generated on XLGB-OA intersection targets. (b) Subnetwork constructed by top 20 targets extracted from PPI network. (c) Top 5 GO items for biological process, cellular component, and molecular function, respectively. (d) Top 10 KEGG pathways sorted in order of *p* values. (e) A rebuilt pathway based on the refining of top KEGG pathways (PPI, protein-protein interaction; XLGB, Xian-Ling-Gu-Bao; OA, osteoarthritis; GO, gene ontology).

PPI network was constructed based on the STRING database, in which the node sizes represented their degree values in the network (Figure 5(a)). Subsequently, the top 20 targets were extracted and then rebuilt to generate a new subnetwork (Figure 5(b)). These targets were ranked in the order of their degree values as follows: TP53, MAPK1, AKT1, STAT3, MAPK3, JUN, RELA, MAPK8, PIK3R1, TNF, and so on. After GO analysis, the top 5 GO items were, respectively, extracted for biological process (BP), cellular component (CC), and molecular function (MF) terms (Figure 5(c)).

To further explore the regulation pathway of XLGB for OA treatment, a pathway-based enrichment analysis was carried out. A total of 155 KEGG pathways were enriched. Among them, the top 10 pathways were extracted based on *p* values, including hepatitis B, Kaposi sarcoma-associated herpesvirus infection, measles, fluid shear stress and atherosclerosis, AGE-RAGE signaling pathway in diabetic complications, apoptosis, toxoplasmosis, TNF signaling pathway, Chagas disease, and colorectal cancer (Figure 5(d)). Among them, two KEGG pathways, named apoptosis and TNF signaling pathway, were assigned to be the most relevant pathways associated with XLGB efficacy on OA. The two pathways were refined and combined, after which one new pathway graphic was rebuilt (Figure 5(e)). In this pathway, the targets labeled with red color belonged to XLGB-OA intersection targets. These labeled targets included JNK, Bcl-2, Bax, CASP 3, PI3K, AKT, NF-*κ*B, IL-6, IL-1*β*, and TNF-*α*, which were key elements in the MAPK signaling pathway and PI3K/AKT/NF-*κ*B signaling pathway. These targets and pathways were deduced to play a pivotal role in the therapeutic effect of XLGB on OA.

### 3.4. Experimental Validation

#### 3.4.1. XLGB Protected ATDC5 Cells against Apoptosis

**Figure 6:**
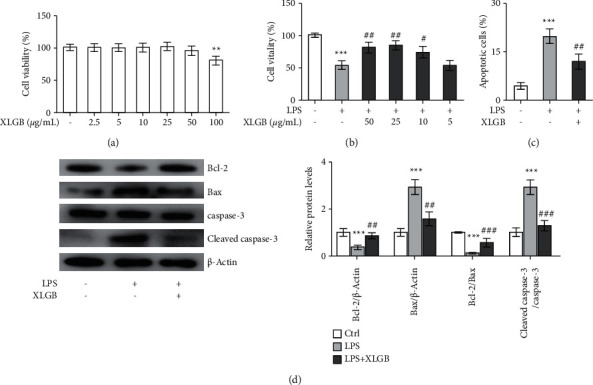
XLGB protected ATDC5 cells against LPS-induced apoptosis. (a) Effects of XLGB on cell viability of chondrocytes. The concentrations of XLGB for treatment ranged from 2.5 to 100 *μ*g/mL. (b) Effects of XLGB on cell viability of LPS-induced chondrocytes. The concentrations of XLGB for treatment ranged from 5 to 50 *μ*g/mL. (c) Effects of XLGB (25 *μ*g/mL) on apoptosis of LPS-induced chondrocytes. (d) Effects of XLGB (25 *μ*g/mL) on the expression of apoptosis-relevant proteins in LPS-induced chondrocytes. ATDC5 cells were treated with 5 *μ*g/mL of LPS for 12 h. For drug administration, the cells were treated with XLGB extracts for 12 h before LPS stimulation. ^*∗*^*p* < 0.05, ^*∗∗*^*p* < 0.01, and ^*∗∗∗*^*p* < 0.001 compared with the control group; ^#^*p* < 0.05, ^##^*p* < 0.01, and ^###^*p* < 0.001 compared with the LPS group (XLGB, Xian-Ling-Gu-Bao; LPS, lipopolysaccharide).

ATDC5 cells were treated with various concentrations of XLGB extract for 72 h, and the viability of cells was tested to evaluate the cytotoxicity of XLGB extract. As shown in Figure 6(a), XLGB extracts with concentrations ≤50 *μ*g/mL did not affect cell viability significantly, whereas XLGB extracts at 100 *μ*g/mL induced a significant reduction in cell viability. Then, XLGB extracts with concentrations ≤50 *μ*g/mL were tested for the effects on LPS-induced cytotoxicity. As shown in Figure 6(b), LPS induced significant cell death in ATDC5 cells as indicated by the decreased cell viability. However, XLGB extracts effectively attenuated LPS-induced cytotoxicity at concentrations of 10–50 *μ*g/mL, and the greatest effect was observed at 25 *μ*g/mL. Thus, 25 *μ*g/mL was selected for use in the following experiments. As shown in Figure 6(c), significant apoptosis was found in LPS-treated cells, which was consistent with the decreased viability. These findings were also coupled with the downregulated expression of Bcl-2 and the upregulated expression of Bax, as well as the cleavage of caspase-3 (Figure 6(d)). Moreover, the ratio of Bcl-2/Bax was significantly decreased in the LPS group, while such a decrease was effectively reversed by XLGB pretreatment (Figure 6(d)). These results suggested that LPS induced significant damage in ATDC5 cells. XLGB pretreatment effectively diminished the LPS-induced apoptosis (Figure 6(c)). Western blot assay also revealed that XLGB extract apparently upregulated Bcl-2 expression, downregulated Bax expression, and prevented caspase-3 cleavage (Figure 6(d)). These data indicated that XLGB could alleviate LPS-induced cytotoxicity and apoptosis in ATDC5 cells.

#### 3.4.2. XLGB Protected ATDC5 Cells against Inflammatory Response

**Figure 7:**
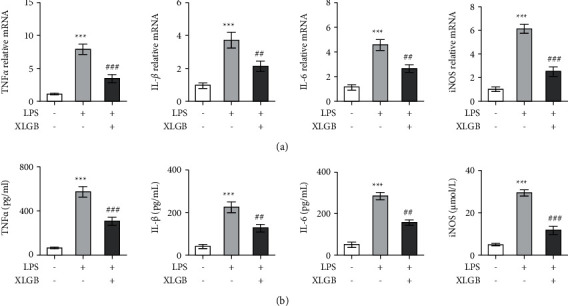
XLGB protected ATDC5 cells against LPS-induced inflammatory response. (a) Effects of XLGB (25 *μ*g/mL) on the expression of TNF-*α*, IL-1*β*, IL-6, and iNOS mRNAs in LPS-induced chondrocytes. (b) Effects of XLGB (25 *μ*g/mL) on the releases of TNF-*α*, IL-1*β*, IL-6, and iNOS from LPS-induced chondrocytes. ATDC5 cells were treated with 5 *μ*g/mL of LPS for 12 h. For drug administration, the cells were treated with XLGB extracts for 12 h before LPS stimulation. ^*∗*^*p* < 0.05, ^*∗∗*^*p* < 0.01, and ^*∗∗∗*^*p* < 0.001 compared with the control group; ^#^*p* < 0.05, ^##^*p* < 0.01, and ^###^*p* < 0.001 compared with the LPS group (XLGB, Xian-Ling-Gu-Bao; LPS, lipopolysaccharide).

The influence of XLGB extract on LPS-induced inflammatory response in ATDC5 cells was evaluated. In comparison with apoptosis results, the same trends were observed in the release of proinflammatory cytokines and iNOS. In LPS-treated cells, the expression of TNF-*α*, IL-1*β*, IL-6, and iNOS was significantly elevated compared to control cells (Figure 7(a)). However, these cytokines and iNOS were significantly downregulated in the LPS + XLGB group in contrast with the LPS group (Figure 7(a)). Likewise, LPS induced significant releases of TNF-*α*, IL-1*β*, IL-6, and iNOS from ATDC5 cells, while the enhanced release of these factors was effectively attenuated by XLGB (Figure 7(b)). Collectively, these results suggested that XLGB effectively relieved LPS-induced inflammatory response in ATDC5 cells.

#### 3.4.3. XLGB Inhibited JNK and PI3K/AKT/NF-*κ*B Signaling Pathways

**Figure 8:**
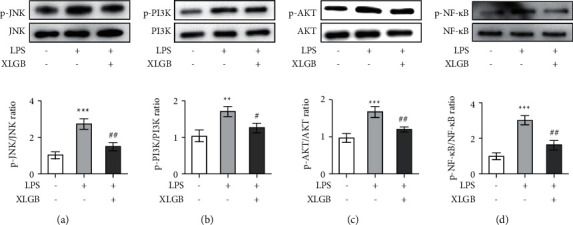
Effects of XLGB (25 *μ*g/mL) on the expression of JNK (a), PI3K (b), AKT (c), and NF-*κ*B (d) in LPS-induced ATDC5 cells. ATDC5 cells were treated with 5 *μ*g/mL of LPS for 12 h. For drug administration, the cells were treated with XLGB extracts for 12 h before LPS stimulation. ^*∗*^*p* < 0.05, ^*∗∗*^*p* < 0.01, and ^*∗∗∗*^*p* < 0.001 compared with the control group; ^#^*p* < 0.05, ^##^*p* < 0.01, and ^###^*p* < 0.001 compared with the LPS group (XLGB, Xian-Ling-Gu-Bao; LPS, lipopolysaccharide).

To further explore the mechanism of XLGB-induced beneficial effects, the expression of key proteins in the JNK and PI3K signaling cascades was examined. Western blot results showed that LPS remarkably enhanced the phosphorylated level of JNK and did not affect the expression level of total JNK (Figure 8(a)), whereas this enhancement of JNK phosphorylation was reversed by XLGB stimulation (Figure 8(a)). In addition, LPS treatment significantly increased the ratios of p/t-PI3K and p/t-AKT, while XLGB pretreatment significantly abolished these LPS-induced increases (Figures 8(b) and 8(c)). Consistently, NF-*κ*B phosphorylation was notably upregulated by LPS treatment compared with the control group, while this upregulation was significantly inhibited by XLGB pretreatment compared with the LPS group (Figure 8(d)). As network analysis deduced that JNK and PI3K signaling cascades played a critical role in XLGB efficacy against OA, these findings from *in vitro* study further confirmed that the amelioration of chondrocyte apoptosis and inflammatory response could have a correlation with the inhibition of JNK, PI3K, AKT, and NF-*κ*B.

## 4. Discussion

The phytochemical constituents are regarded as the cornerstone of herbal medicine; thus, precise constituent verification is critical for following network analysis and mechanism deduction. In this work, phytochemical components in XLGB capsules were detected by an LC-MS method. As a result, a total of 55 compounds were identified as phenolic acids, flavonoid glycosides, pyranocoumarins, and tanshinones. These results were consistent with the data from previous reports [19, 27]. According to their affiliation to herbs, these compounds mainly belonged to CcM, SmB, and EbM. In addition, CcM, SmB, and EbM yielded plenty of targets and contributed a lot to XLGB targets, as well as intersection targets between XLGB and OA. However, SmB possessed relatively low similarity with OA regarding target profile, although it had the most targets among the total of 6 herbs. By contrast, EbM and CcM showed the most similar target profile to XLGB and OA. Overall, EbM and CcM played an important role and had a pivotal position in the XLGB formula, which was roughly in line with the positions of EbM as the king drug and CcM as the minister drug in the XLGB recipe.

The knowledge about OA pathogenesis is still evolving. Currently, OA is considered a whole-organ structural disease involving complex and multifactorial pathophysiology [5, 28]. It has been demonstrated that a wide range of inflammatory components, mechanical overload, metabolic alterations, and cell senescence lead to similar outcomes of joint destruction in OA [29–31]. Joint inflammation is common in OA, involving cytokines, chemokines, and inflammatory mediators, which contribute to the progressive joint damage and pain in OA [6]. For detail, factors involved in the inflammatory state include IL-1*β*, IL-6, TNF-*α*, PGE2, and iNOS. In addition, cell death with morphological and molecular features of apoptosis occurs in OA cartilage [32]. Chondrocyte death and matrix loss may aggravate each other to form a vicious cycle, suggesting that there is a definite correlation between the degree of cartilage damage and chondrocyte apoptosis [32]. Therefore, inflammatory factors and chondrocyte apoptosis play crucial roles in OA pathogenesis and also represent the potential targets to modulate cartilage degeneration.

XLGB formula consisting of 6 medicinal herbs has a clinical practice in OA treatment for a long time [15, 16]. It was believed that its therapeutic efficacy could be derived from the interaction of multiple constituents regulating multiple targets and signaling pathways. Based on network analysis, core active constituents including icariin, magnoflorine, tanshinone IIA, psoralidin, and bavachin were considered as the main material basis accounting for the efficacy of XLGB against OA. Meanwhile, a series of potential targets and pathways were proposed, which were considered as the pivotal action mechanism of XLGB for OA treatment. For detail, these targets included MAPKs, PI3K, AKT, BAX, BCL2, CASP3, RELA, IL1B, TNF, and NOS2. These targets are closely involved in chondrocyte apoptosis, synovium inflammation, and cartilage destruction, which could be potential targets for OA treatment [21, 32, 33]. Indeed, several studies demonstrated that XLGB and its monarch herb inhibited the apoptosis of cartilage tissue cells and decreased cytokine levels, including IL-1*β*, IL-6, and TNF-*α* [34, 35]. Accordingly, our *in vitro* study demonstrated that XLGB effectively attenuated LPS-induced cytotoxicity and apoptosis in ATDC5 cells, along with downregulation of Bcl-2 and cleaved caspase-3, as well as upregulation of Bax. Besides, our *in vitro* study also indicated that XLGB reduced the expression of IL-1*β*, IL-6, and TNF-*α* in LPS-induced ATDC5 cells by inhibiting NF-*κ*B phosphorylation.

Based on pathway enrichment and refining, the JNK signaling pathway and PI3K/AKT/NF-*κ*B signaling pathway were deduced as the most promising regulation pathways of XLGB for the treatment of OA. JNK is a member of the MAPK superfamily, and phosphorylated (activated) JNK could mediate antiapoptotic proteins Bcl-2/Bcl-xL phosphorylation to induce cytochrome C, caspase-9, caspase-3 apoptosis cascade [36]. As validation, our *in vitro study* indicated that XLGB pretreatment remarkably abolished the LPS-induced decline of the Bcl-2/Bax ratio and caspase-3 cleaving. These beneficial effects could be attributed to the inhabitation of JNK activation. PI3K/AKT pathway plays a central role in regulating inflammatory responses, while NF-*κ*B pathway is generally acknowledged as a prototypical proinflammatory pathway. It is generally agreed that PI3K/Akt pathway could be an upstream activator of the NF-*κ*B signaling cascade, thus emerging as a therapeutic target for inflammatory diseases [37–39]. Therefore, it was deduced that regulation of PI3K/AKT/NF-*κ*B signaling pathway could be a pivotal action mechanism of XLGB for managing inflammation in OA. In order to validate this notion, the phosphorylation levels of PI3K, AKT, and NF-*κ*B were detected in ATDC5 cells. Interestingly, XLGB treatment apparently reversed the phosphorylation of these proteins. Hence, our data suggested that XLGB alleviated LPS-induced inflammatory response partly by suppressing NF-*κ*B phosphorylation which might be related to the inhibition of PI3K/Akt. Collectively, our work suggested that the efficacy of XLGB was involved in the inhibition of chondrocyte apoptosis and inflammatory response, and the mechanism was supposed to the inhibition of JNK and PI3K/Akt/NF-*κ*B signaling pathways.

## 5. Conclusion

To summarize, our work aimed at elucidating the medicinal material basis and mechanism of XLGB for OA treatment. The phytochemical constituents were detected in XLGB capsules serving as the cornerstone of following network analysis. Based on target analysis, EbM and CcM presented a very similar target profile to XLGB and OA among the herbs in XLGB, which was roughly in line with the positions of EbM and CcM as the king and minister drugs, respectively, in the XLGB recipe. Through network analysis, the core targets of XLGB were presumed to be MAPKs, PI3K, AKT, BCL2, RELA, TNF, NOS2, and so on, and the mechanism was speculated to mainly inhibit chondrocyte apoptosis and inflammatory response through JNK and PI3K signaling cascade. Consistently, the *in vitro* results confirmed that XLGB effectively attenuated LPS-induced apoptosis and inflammation in ATDC5 cells, and the mechanism was supposed to the inhibition of JNK and PI3K/AKT/NF-*κ*B signaling pathways. Our study could provide a scientific basis for further research and clinical use of XLGB capsule.

## Figures and Tables

**Table 1:** Primer sequence.

Gene	Forward	Reverse
TNF-*α*	5′-GCTGCACTTTGGAGTGATCG-3′	5′-CTTGTCACTCGGGGTTCGAG-3′
IL-1*β*	5′-TGGACCTTCCAGGATGAGGACA-3′	5′-GTTCATCTCGGAGCCTGTAGTG-3′
IL-6	5′-TTCGGTCCAGTTGCCTTCTC-3′	5′-TCTTCTCCTGGGGGTACTGG-3′
iNOS	5′-CACCAAGCTGAACTTGAGCG-3′	5′-CGTGGCTTTGGGCTCCTC-3′
*β*-Actin	5′-GTACGCCAACACAGTGCTG-3′	5′-CGTCATACTCCTGCTTGCTG-3′

## Data Availability

The data used to support the findings of this study are included within the article.

## Supplementary Materials

Supplementary Materials Supplementary Table S1. Compounds identified in the extract of XLGB.
