# Supplementary material for: Multitech-Based Study on Medicinal Material Basis and Action Mechanism of Herbal Formula Xian-Ling-Gu-Bao Capsule in Treatment of Osteoarthritis
Source: Evid Based Complement Alternat Med. 2022 Sep 6;2022:6986372. doi: 10.1155/2022/6986372 (PMC9470326; doi:10.1155/2022/6986372)
Supplement: Supplementary Materials — Supplementary Table S1. Compounds identified in the extract of XLGB. [file 6986372.f1.docx]

Supplementary Material

Table S1 Compounds identified in the extract of XLGB

| No. | RT (min) | Identification | Precursor | Calculated mass  (m/z) | Measured mass  (m/z) | Error (ppm) | Formula |
| --- | --- | --- | --- | --- | --- | --- | --- |
| 1 | 1.5 | Caffeic acid | [M-H]^-^ | 179.0344 | 179.0328 | 8.9 | C_9_H_8_O_4_ |
| 2 | 1.8 | Salidroside | [M-H]^-^ | 299.1130 | 299.1149 | -6.4 | C_14_H_20_O_7_ |
| 3 | 3.1 | Ferulic acid | [M-H]^-^ | 193.0500 | 193.0485 | 7.8 | C_10_H_10_O_4_ |
| 4 | 3.9 | Loganic acid | [M-H]^-^ | 375.1290 | 375.1276 | 3.7 | C_16_H_24_O_10_ |
| 5 | 4.9 | cis-3-Caffeoylquinic acid | [M-H]^-^ | 353.1024 | 353.1039 | -4.2 | C_16_H_18_O_9_ |
| 6 | 5.8 | Eurycarpin A | [M-H]^-^ | 337.1075 | 337.1091 | -4.7 | C_20_H_18_O_5_ |
| 7 | 6.2 | Magnoflorine | [M+H]^+^ | 343.1784 | 343.1767 | 5.0 | C_20_H_24_NO_4_^+^ |
| 8 | 6.6 | Psoralenoside | [M-H]^-^ | 365.0872 | 365.0849 | 6.3 | C_17_H_18_O_9_ |
| 9 | 6.8 | Isopsoralenoside | [M-H]^-^ | 365.0872 | 365.0853 | 5.2 | C_17_H_18_O_9_ |
| 10 | 7.0 | Sweroside | [M+H]^+^ | 359.1342 | 359.1322 | 5.6 | C_16_H_22_O_9_ |
| 11 | 8.0 | Cycloolivil | [M+H]^+^ | 377.1601 | 377.1618 | -4.5 | C_20_H_24_O_7_ |
| 12 | 9.0 | 3,4-Dicaffeoylquinic acid | [M-H]^-^ | 515.1188 | 515.1162 | 5.0 | C_25_H_24_O_12_ |
| 13 | 9.4 | Timosaponin BII | [M-H]^-^ | 920.4981 | 920.5012 | -3.4 | C_45_H_76_O_19_ |
| 14 | 9.1 | Epimedoside C | [M-H]^-^ | 515.1552 | 515.1531 | 4.1 | C_26_H_28_O_11_ |
| 15 | 9.5 | Corylifol B | [M+H]^+^ | 341.1389 | 341.1405 | -4.7 | C_20_H_20_O_5_ |
| 16 | 9.9 | Salvianolic acid B | [M-H]^-^ | 717.6058 | 717.6027 | 4.3 | C_36_H_30_O_16_ |
| 17 | 11.4 | Icariin | [M-H]^-^ | 675.2288 | 675.2316 | -4.1 | C_33_H_40_O_15_ |
| 18 | 11.4 | Des-O-methylicariin | [M-H]^-^ | 661.6272 | 661.6249 | 3.5 | C_32_H_38_O_15_ |
| 19 | 11.4 | Epimedin C | [M+H]^+^ | 823.3025 | 823.3057 | -3.9 | C_39_H_50_O_19_ |
| 20 | 11.8 | Epimedin B | [M-H]^-^ | 807.2710 | 807.2735 | -3.1 | C_38_H_48_O_19_ |
| 21 | 11.8 | Anhydroicaritin-3-O-rhamnopyranoside | [M-H]^-^ | 513.1759 | 513.1736 | 4.5 | C_27_H_30_O_10_ |
| 22 | 11.7 | Icariside I | [M+H]^+^ | 531.1867 | 531.1849 | 3.4 | C_27_H_30_O_11_ |
| 23 | 12.6 | Cistanoside F | [M-H]^-^ | 487.1450 | 487.1429 | 4.3 | C_21_H_28_O_13_ |
| 24 | 12.7 | Neogitogenin | [M+H]^+^ | 433.3318 | 433.3337 | -4.4 | C_27_H_44_O_4_ |
| 25 | 12.8 | Macranthoside A | [M-H]^-^ | 912.0882 | 912.0861 | 2.3 | C_47_H_76_O_17_ |
| 26 | 13.3 | Paramiltioic acid | [M-H]^-^ | 331.3829 | 331.3846 | -5.1 | C_19_H_24_O_5_ |
| 27 | 13.3 | Chlorogenic acid | [M+H]^+^ | 355.1029 | 355.1045 | -4.5 | C_16_H_18_O_9_ |
| 28 | 13.5 | Psoralen | [M+H]^+^ | 187.0396 | 187.0382 | 7.5 | C_11_H_6_O_3_ |
| 29 | 13.9 | Sagittatoside B | [M-H]^-^ | 645.2182 | 645.2154 | 4.3 | C_32_H_38_O_14_ |
| 30 | 14.0 | Isopsoralen | [M+H]^+^ | 187.0396 | 187.0381 | 8.0 | C_11_H_6_O_3_ |
| 31 | 14.8 | Neochlorogenic acid | [M+H]^+^ | 355.1029 | 355.1046 | -4.8 | C_16_H_18_O_9_ |
| 32 | 14.8 | Ikarisoside A | [M-H]^-^ | 499.1603 | 499.1587 | 3.2 | C_26_H_28_O_10_ |
| 33 | 15.9 | Hexandraside E | [M-H]^-^ | 677.2080 | 677.2049 | 4.6 | C_32_H_38_O_16_ |
| 34 | 16.9 | Ikarisoside B | [M-H]^-^ | 661.2131 | 661.2112 | 2.9 | C_32_H_38_O_15_ |
| 35 | 16.9 | Anhydroicaritin | [M-H]^-^ | 367.1180 | 367.1163 | 4.6 | C_21_H_20_O_6_ |
| 36 | 17.3 | Isobavachin | [M-H]^-^ | 323.1282 | 323.1268 | 4.3 | C_20_H_20_O_4_ |
| 37 | 18.3 | Icaritin | [M+H]^+^ | 369.1338 | 369.1321 | 4.6 | C_21_H_20_O_6_ |
| 38 | 18.4 | Baohuoside I | [M-H]^-^ | 513.1759 | 513.1731 | 5.5 | C_27_H_30_O_10_ |
| 39 | 18.6 | Neobavaisoflavone | [M-H]^-^ | 321.1126 | 321.1144 | -5.6 | C_20_H_18_O_4_ |
| 40 | 19.3 | Bavachin | [M-H]^-^ | 323.1282 | 323.1268 | 4.3 | C_20_H_20_O_4_ |
| 41 | 19.9 | Epimedoside A | [M-H]^-^ | 661.2131 | 661.2156 | -3.8 | C_32_H_38_O_15_ |
| 42 | 21.1 | Corylin | [M-H]^-^ | 319.0969 | 319.0983 | -4.4 | C_20_H_16_O_4_ |
| 43 | 21.7 | Yinyanghuo C | [M-H]^-^ | 335.0918 | 335.0939 | -6.3 | C_20_H_16_O_5_ |
| 44 | 21.8 | Psoralidin | [M+H]^+^ | 336.0997 | 336.0983 | 4.2 | C_20_H_16_O_5_ |
| 45 | 22.4 | Dihydrotanshinone | [M+H]^+^ | 279.1021 | 279.1005 | 5.7 | C_18_H_14_O_3_ |
| 46 | 23.7 | Bavachalcone | [M-H]^-^ | 323.1282 | 323.1267 | 4.6 | C_20_H_20_O_4_ |
| 47 | 24.2 | Isotanshinone IIA | [M+H]^+^ | 295.1334 | 295.1315 | 6.4 | C_19_H_18_O_3_ |
| 48 | 24.7 | Bavachinin | [M+H]^+^ | 339.1597 | 339.1611 | -4.1 | C_21_H_22_O_4_ |
| 49 | 25.5 | Corylifol A | [M-H]^-^ | 389.1752 | 389.1771 | -4.9 | C_25_H_26_O_4_ |
| 50 | 25.8 | Cryptotanshinone | [M+H]^+^ | 297.1491 | 297.1504 | -4.4 | C_19_H_20_O_3_ |
| 51 | 27.5 | Methyltanshinonate | [M+H]^+^ | 339.1233 | 339.1217 | 4.7 | C_20_H_18_O_5_ |
| 52 | 27.8 | Damascenone | [M+H]^+^ | 191.1436 | 191.1421 | 7.8 | C_13_H_18_O |
| 53 | 27.5 | Danshenol A | [M-H]^-^ | 335.1282 | 335.1267 | 4.5 | C_21_H_20_O_4_ |
| 54 | 29.1 | Tanshinone IIA | [M+H]^+^ | 295.1334 | 295.1318 | 5.4 | C_19_H_18_O_3_ |
| 55 | 30.2 | Isotanshinone IIB | [M+H]^+^ | 311.1284 | 311.1272 | 3.9 | C_19_H_18_O_4_ |
